# Ethane-1,2-diammonium dibromide: a redetermination at 100 K

**DOI:** 10.1107/S1600536810033313

**Published:** 2010-08-28

**Authors:** Charmaine Arderne, Gert J. Kruger

**Affiliations:** a University of Johannesburg, Department of Chemistry, PO Box 524, Auckland Park, Johannesburg 2006, South Africa

## Abstract

In the redetermined [for the previous study, see Søtofte (1976 ▶). *Acta Chem. Scand. Ser. A*, **30**, 309–311] crystal structure of the title compound, C_2_H_10_N_2_^2+^·2Br^−^, the H atoms have been located and the hydrogen-bonding scheme is described. The ethane-1,2-diammonium cation lies over a crystallographic inversion centre and straddles a crystallographic mirror plane with the C and N atoms in special positions. In the crystal, the cations and anions are linked by N—H⋯Br and N—H⋯(Br,Br) hydrogen bonds, which generate various ring and chain motifs including an *R*_10_^5^(32) loop.

## Related literature

For the previous structure, see: Søtofte (1976 ▶). For hydrogen-bond motifs, see: Bernstein *et al.* (1995 ▶). For information on the Cambridge Database, see: Allen (2002 ▶).
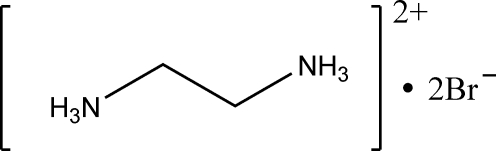


## Experimental

### 

#### Crystal data

C_2_H_10_N_2_^2+^·2Br^−^
*M* *_r_* = 221.94
Monoclinic, 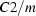

*a* = 15.144 (2) Å
*b* = 4.7598 (7) Å
*c* = 4.8146 (7) Å
β = 101.323 (2)°
*V* = 340.30 (8) Å^3^
*Z* = 2
Mo *K*α radiation
μ = 11.80 mm^−1^
*T* = 100 K
0.36 × 0.24 × 0.20 mm

#### Data collection

Bruker APEXII CCD diffractometer
Absorption correction: multi-scan (AXScale; Bruker, 2010 ▶) *T*_min_ = 0.101, *T*_max_ = 0.201
3261 measured reflections
481 independent reflections
475 reflections with *I* > 2σ(*I*)
*R*_int_ = 0.024

#### Refinement

*R*[*F* ^2^ > 2σ(*F* ^2^)] = 0.013
*wR*(*F* ^2^) = 0.035
*S* = 1.17
481 reflections
28 parameters
H atoms treated by a mixture of independent and constrained refinement
Δρ_max_ = 0.62 e Å^−3^
Δρ_min_ = −0.39 e Å^−3^

### 

Data collection: *APEX2* (Bruker, 2010 ▶); cell refinement: *SAINT* (Bruker, 2010 ▶); data reduction: *SAINT*; program(s) used to solve structure: *SHELXS97* (Sheldrick, 2008 ▶); program(s) used to refine structure: *SHELXL97* (Sheldrick, 2008 ▶); molecular graphics: *X-SEED* (Barbour, 2001 ▶) and *Mercury* (Macrae *et al.*, 2006 ▶).; software used to prepare material for publication: *publCIF* (Westrip, 2010 ▶) and *PLATON* (Spek, 2009 ▶).

## Supplementary Material

Crystal structure: contains datablocks I, global. DOI: 10.1107/S1600536810033313/hb5586sup1.cif

Structure factors: contains datablocks I. DOI: 10.1107/S1600536810033313/hb5586Isup2.hkl

Additional supplementary materials:  crystallographic information; 3D view; checkCIF report

## Figures and Tables

**Table 1:** Hydrogen-bond geometry (Å, °)

*D*—H⋯*A*	*D*—H	H⋯*A*	*D*⋯*A*	*D*—H⋯*A*
N1—H2*A*⋯Br1	0.83 (4)	2.89 (3)	3.324 (2)	115 (3)
N1—H2*A*⋯Br1^i^	0.83 (4)	3.00 (2)	3.4808 (14)	120 (1)
N1—H2*B*⋯Br1^ii^	0.88 (2)	2.48 (2)	3.3326 (14)	163 (2)

Symmetry codes: (i) 
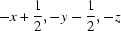
; (ii) 
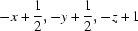
.

**Fig. 1.**
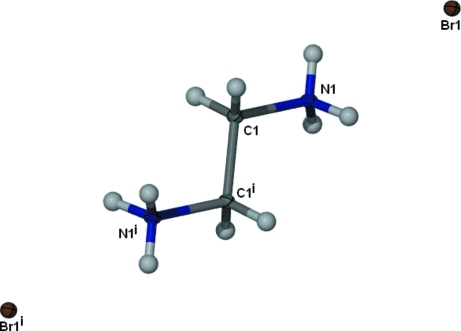
: Molecular structure of (I), with displacement ellipsoids drawn at the 50% probability level. Atoms labelled with (i) are at the symmetry position (1 - x, y, 1 - z)

**Fig. 2.**
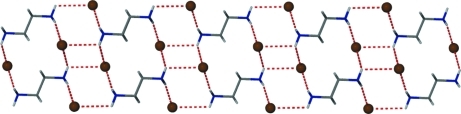
: Packing arrangement if (I) viewed down the b axis and rotated slightly. Hydrogen bonds are indicated by dashed lines.

**Fig. 3.**
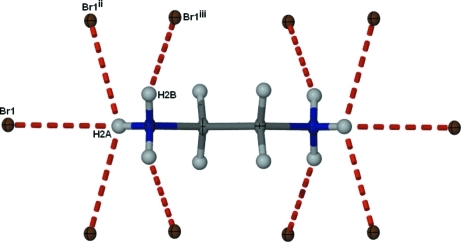
: Close-up view of (I) viewed down the b axis with a slight offset clearly showing the hydrogen-bonding interactions. Hydrogen bonds are indicated by dashed lines.

**Fig. 4.**
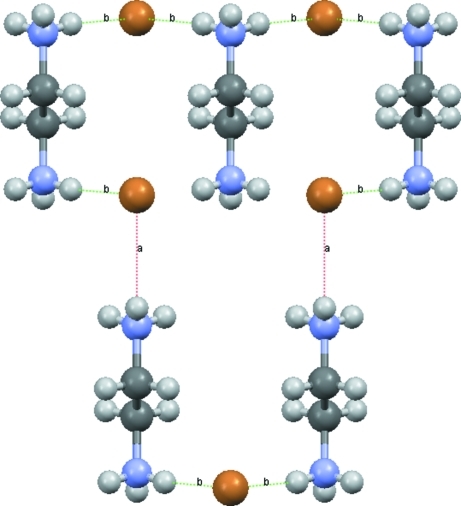
: Close up view of (I) viewed down the c axis showing the T-shaped ring motif involving five diammonium cations and five bromide anions.

## supplementary crystallographic information

### Comment

As part of our ongoing study of the structural characteristics of organic-inorganic layered diammonium salts, the crystal structure of ethane-1,2-diammonium dibromide, (I), was determined. A search of the Cambridge Structural Database (Version 5.31, May 2010 release; Allen, 2002) revealed that the crystal structure of (I) had been previously determined 34 years ago (Søtofte, 1976) at room temperature. The information in the CSD CIF file however appears incomplete and the author also states that the contributions from the hydrogen atoms in the structure was ignored. Here we report the redetermined structure of the title compound at 100 K. All the H atom positions were clearly visible in the difference Fourier map and they were independently refined with ADP's constrained to values of 1.2 and 1.5 times the isotropic U values of the C and N atoms on which they ride. We also show packing arrangements, hydrogen bonding interactions, hydrogen bonding motifs as well as calculated torsion angles (Table 3) that were previously not reported.

The ethane-1,2-diammonium cation lies over a centre of inversion and also straddles a mirror plane. The asymmetric unit contains one bromide anion and half of the ethane-1,2-diammonium cation (Figure 1).

Figure 2 illustrates the packing of the title compound viewed down the *b* axis. The ethane-1,2-diammonium cations are stacked above one another in the *ac* plane linked together by hydrogen bonds.

A close-up view of the hydrogen bonding interactions can be viewed in Figure 3. The hydrogen bond distances and angles for (I) can be found in Table 2. The hydrogen bonding network is three-dimensional and particularly complex, consisting of a variety of ring and chain motifs (identified using graphs sets in Mercury (Macrae *et al.*, 2006). Because of the complexity and number of different motifs identified, we focus on one particularly interesting hydrogen-bonding ring motif in the structure that appears to be in the shape of a T (Figure 4.) and it was chosen to best describe the highest level hydrogen bonding motif evident in the crystal structure.

Figure 4 shows a view of five diammonium cations and five bromide anions (viewed down the *c* axis) that are hydrogen bonded together to form a large, 32-membered T-shaped ring motif with graph set notation *R*^5^_10_(32). Other ring motifs are evident - eight ring motifs and four chain motifs were identified from Mercury (Macrae *et al.*, 2006) but are not depicted here.

### Experimental

Compound (I) was prepared by adding 1,2-diamino-ethane (0.50 g, 2.25 mmol) to 47% hydrobromic acid (HBr, 2 ml, 37.07 mmol, Merck) in a sample vial. The mixture was then refluxed at 363 K for 2 h. The solution was cooled at 2 K h^-1^ to room temperature. Colourless blocks of (I) were collected.

### Refinement

H atoms were clearly visible from the difference Fourier map. They were independently refined with the constraints *U*_iso_(H) = 1.2Ueq(C) and 1.5Ueq(N). For (I), the highest peak in the final difference map is 0.80Å from Br1 and the deepest hole is 0.98Å from C1.

### Crystal data

**Table:**

C_2_H_10_N_2_^2+^·2Br^−^	*F*(000) = 212
*M**_r_* = 221.94	*D*_x_ = 2.166 Mg m^−^^3^
Monoclinic, *C*2/*m*	Mo *K*α radiation, λ = 0.71073 Å
Hall symbol: -C 2y	Cell parameters from 2937 reflections
*a* = 15.144 (2) Å	θ = 2.7–28.4°
*b* = 4.7598 (7) Å	µ = 11.80 mm^−^^1^
*c* = 4.8146 (7) Å	*T* = 100 K
β = 101.323 (2)°	Block, colourless
*V* = 340.30 (8) Å^3^	0.36 × 0.24 × 0.20 mm
*Z* = 2	

### Data collection

**Table:**

Bruker APEXII CCD diffractometer	481 independent reflections
Radiation source: fine-focus sealed tube	475 reflections with *I* > 2σ(*I*)
graphite	*R*_int_ = 0.024
φ and ω scans	θ_max_ = 28.4°, θ_min_ = 2.7°
Absorption correction: multi-scan (AXScale; Bruker, 2010)	*h* = −20→20
*T*_min_ = 0.101, *T*_max_ = 0.201	*k* = −6→6
3261 measured reflections	*l* = −6→6

### Refinement

**Table:**

Refinement on *F*^2^	Secondary atom site location: difference Fourier map
Least-squares matrix: full	Hydrogen site location: difference Fourier map
*R*[*F*^2^ > 2σ(*F*^2^)] = 0.013	H atoms treated by a mixture of independent and constrained refinement
*wR*(*F*^2^) = 0.035	*w* = 1/[σ^2^(*F*_o_^2^) + (0.0165*P*)^2^ + 0.5674*P*] where *P* = (*F*_o_^2^ + 2*F*_c_^2^)/3
*S* = 1.17	(Δ/σ)_max_ < 0.001
481 reflections	Δρ_max_ = 0.62 e Å^−^^3^
28 parameters	Δρ_min_ = −0.39 e Å^−^^3^
0 restraints	Extinction correction: *SHELXL97* (Sheldrick, 2008), Fc^*^=kFc[1+0.001xFc^2^λ^3^/sin(2θ)]^-1/4^
Primary atom site location: structure-invariant direct methods	Extinction coefficient: 0.051 (3)

### Special details

**Table:**

Geometry. All e.s.d.'s (except the e.s.d. in the dihedral angle between two l.s. planes) are estimated using the full covariance matrix. The cell e.s.d.'s are taken into account individually in the estimation of e.s.d.'s in distances, angles and torsion angles; correlations between e.s.d.'s in cell parameters are only used when they are defined by crystal symmetry. An approximate (isotropic) treatment of cell e.s.d.'s is used for estimating e.s.d.'s involving l.s. planes.
Refinement. Refinement of *F*^2^ against ALL reflections. The weighted *R*-factor *wR* and goodness of fit *S* are based on *F*^2^, conventional *R*-factors *R* are based on *F*, with *F* set to zero for negative *F*^2^. The threshold expression of *F*^2^ > σ(*F*^2^) is used only for calculating *R*-factors(gt) *etc*. and is not relevant to the choice of reflections for refinement. *R*-factors based on *F*^2^ are statistically about twice as large as those based on *F*, and *R*- factors based on ALL data will be even larger.

### Fractional atomic coordinates and isotropic or equivalent isotropic displacement parameters (Å^2^)

**Table:**

	*x*	*y*	*z*	*U*_iso_*/*U*_eq_	
C1	0.47174 (14)	0.0000	0.3516 (4)	0.0119 (4)	
N1	0.37420 (13)	0.0000	0.3658 (4)	0.0113 (4)	
Br1	0.151104 (12)	0.0000	0.17162 (4)	0.01051 (13)	
H1A	0.4815 (14)	0.166 (5)	0.246 (4)	0.013*	
H2A	0.344 (2)	0.0000	0.203 (8)	0.016*	
H2B	0.3589 (15)	0.147 (5)	0.457 (5)	0.016*	

### Atomic displacement parameters (Å^2^)

**Table:**

	*U*^11^	*U*^22^	*U*^33^	*U*^12^	*U*^13^	*U*^23^
C1	0.0081 (9)	0.0184 (11)	0.0088 (9)	0.000	0.0007 (7)	0.000
N1	0.0097 (8)	0.0157 (9)	0.0079 (8)	0.000	0.0001 (7)	0.000
Br1	0.00972 (15)	0.01224 (16)	0.00940 (15)	0.000	0.00147 (8)	0.000

### Geometric parameters (Å, °)

**Table:**

C1—N1	1.492 (3)	N1—H2A	0.83 (4)
C1—C1^i^	1.515 (4)	N1—H2B	0.88 (2)
C1—H1A	0.97 (2)		
			
N1—C1—C1^i^	109.8 (2)	C1—N1—H2A	109 (2)
N1—C1—H1A	106.2 (12)	C1—N1—H2B	112.5 (14)
C1^i^—C1—H1A	112.3 (12)	H2A—N1—H2B	108.7 (19)

Symmetry codes: (i) −*x*+1, −*y*, −*z*+1.

### Hydrogen-bond geometry (Å, °)

**Table:**

*D*—H···*A*	*D*—H	H···*A*	*D*···*A*	*D*—H···*A*
N1—H2A···Br1	0.83 (4)	2.89 (3)	3.324 (2)	115 (3)
N1—H2A···Br1^ii^	0.83 (4)	3.00 (2)	3.4808 (14)	120.(1)
N1—H2B···Br1^iii^	0.88 (2)	2.48 (2)	3.3326 (14)	163 (2)

Symmetry codes: (ii) −*x*+1/2, −*y*−1/2, −*z*; (iii) −*x*+1/2, −*y*+1/2, −*z*+1.
